# Maintaining Genome Integrity during Seed Development in *Phaseolus vulgaris* L.: Evidence from a Transcriptomic Profiling Study

**DOI:** 10.3390/genes9100463

**Published:** 2018-09-20

**Authors:** José Ricardo Parreira, Alma Balestrazzi, Pedro Fevereiro, Susana de Sousa Araújo

**Affiliations:** 1 Plant Cell Biotechnology Laboratory, Instituto de Tecnologia Química e Biológica António Xavier, Universidade Nova de Lisboa, Av. da República, 2780-157 Oeiras, Portugal; jsalvado@itqb.unl.pt (J.R.P.); psalema@itqb.unl.pt (P.F.); 2 Plant Biotechnology Laboratory, Department of Biology and Biotechnology “Lazzaro Spallanzani”, University of Pavia, via Ferrata 1, 27100 Pavia, Italy; alma.balestrazzi@unipv.it; 3 Departamento de Biologia Vegetal, Faculdade de Ciências da Universidade e Lisboa, Campo Grande, 1749-016 Lisboa, Portugal

**Keywords:** genome integrity, DNA damage response, chromatin remodeling, *Phaseolus vulgaris*, seed development

## Abstract

The maintenance of genome integrity is crucial in seeds, due to the constant challenge of several endogenous and exogenous factors. The knowledge concerning DNA damage response and chromatin remodeling during seed development is still scarce, especially in *Phaseolus vulgaris* L. A transcriptomic profiling of the expression of genes related to DNA damage response/chromatin remodeling mechanisms was performed in *P. vulgaris* seeds at four distinct developmental stages, spanning from late embryogenesis to seed desiccation. Of the 14,001 expressed genes identified using massive analysis of cDNA ends, 301 belong to the DNA MapMan category. In late embryogenesis, a high expression of genes related to DNA damage sensing and repair suggests there is a tight control of DNA integrity. At the end of filling and the onset of seed dehydration, the upregulation of genes implicated in sensing of DNA double-strand breaks suggests that genome integrity is challenged. The expression of chromatin remodelers seems to imply a concomitant action of chromatin remodeling with DNA repair machinery, maintaining genome stability. The expression of genes related to nucleotide excision repair and chromatin structure is evidenced during the desiccation stage. An overview of the genes involved in DNA damage response and chromatin remodeling during *P. vulgaris* seed development is presented, providing insights into the mechanisms used by developing seeds to cope with DNA damage.

## 1. Introduction

Maintenance of genome integrity is particularly important to the seed phase of the plant lifecycle [1]. Chromatin integrity is constantly being challenged by environmental factors like drought and ionizing radiation or free radicals and alkylating agents generated by endogenous processes [2,3]. These agents cause a variety of DNA damage, including DNA base oxidation and alkylation, the formation of pyrimidine dimers and abasic sites, single- and double-strand breaks (SSBs and DSBs), and DNA inter-strand crosslinks, and therefore seriously threaten the integrity of the plant genome [2]. To maintain genome stability, all organisms have evolved DNA Damage Response (DDR) mechanisms that activate cell cycle checkpoints and DNA repair pathways and programmed cell death [4]. 

Two signal transducers of DNA breakage are the ATAXIA TELANGIECTASIA MUTATED (ATM) and ATM- AND RAD3-RELATED (ATR) kinases [5]. While ATR seems to be critical in the response to disturbances during the progression of DNA replication, the ATM kinase is activated in response to double-strand breaks (DSBs) [6]. The MRE11-RAD50-NBS1 (MRN) complex and REPLICATION PROTEIN A (RPA) complexes sense the damage and trigger activation of the ATM and ATR kinases. This leads to the transcription of the WEE1 kinase, triggering the intra-S checkpoint, and to the phosphorylation of SUPPRESSOR OF GAMMA RESPONSE 1 (SOG1), which induces the transcription of DDR genes [7,8,9]. Downstream of the sensing mechanism, several DNA repair mechanisms can act, including base excision repair (BER), the nucleotide excision repair (NER), the mismatch repair (MMR), and double-strand break (DSB) repair [4]. Nevertheless, to detect and repair damaged DNA, DDR proteins need to overcome the barrier of condensed chromatin to gain access to the lesion in the DNA [10]. Multiple studies in eukaryotes, including plants, have evidenced a strong link between DDR and chromatin structure stability [10,11,12,13].

DNA lesions must be repaired to avoid cytotoxic and genotoxic consequences that adversely affect plant growth and development [14]. Seed germination has been widely exploited as a biological system to study DDR in plants. Effective DNA repair during early germination steps, such as imbibition, has been linked to enhanced seed vigor [14], while other works highlight the role of DDR in maintaining seed longevity and viability [1,15]. While genome integrity mechanisms have been extensively characterized during seed germination, the current knowledge concerning the link between DDR and chromatin remodeling during seed development (SD) is still scarce. Embryogenesis, with a high rate of cell division, as well as desiccation, in which orthodox seeds lose water while maintaining viability, are developmental stages prone to DNA damage. Indeed, a significant proportion of the gene expression and metabolic signatures of seed desiccation resemble those associated with seed germination, implying that seeds already start to be equipped for germination during desiccation [16]. Consequently, disturbances during seed development may impact subsequent seed physiology, as desiccation, dormancy, longevity, and germination [17]. As an example, an essential role for the apurinic/apyrimidinic endonucleases APE1L and APE2, involved in DNA repair during embryo development has been described in *Arabidopsis* [18]. Also, a differential accumulation of DNA repair-related proteins, such as RAD23-like protein, has been described during seed development of *Brassica campestris* L. [19]. Nevertheless, more studies targeting different phenological stages are needed to provide a comprehensive picture of the different DDR mechanisms activated to ensure the maintenance of genome integrity in seeds.

The common bean (*Phaseolus vulgaris* L.) is among the most important grain legumes for human consumption worldwide [20]. Recently, our team unveiled new clues about the maintenance of genome integrity/DDR during *P. vulgaris* seed development, such as the differential accumulation of the PROLIFERATING CELL NUCLEAR ANTIGEN (PCNA) and DNA-DAMAGE INDUCIBLE PROTEIN 1, using a proteomic approach [21]. Genome integrity is, however, maintained as a result of intricate actions of multiple players and an extended characterization of DDR/chromatin remodeling mechanisms occurring during seed development in this species needs to be accomplished. 

To extend our knowledge on this topic, we conducted a transcriptome study to explore the transcriptomic landscape of the DDR/chromatin remodeling mechanisms activated during SD in *P. vulgaris*. Our research hypothesis assumes that the expression of genes involved in DDR is regulated during SD to maintain genome integrity and seed viability upon desiccation. Our study, conducted at four distinct SD time points, presents an overview of the genes involved in the timeframe of these processes while providing new insights into the mechanisms that seeds likely use to cope with DNA damage during development.

## 2. Materials and Methods

### 2.1. Plant Material

Plants from the *Phaseolus vulgaris* genotype SER 16 (SER), kindly supplied by Dr. Steve Beebe of the International Center for Tropical Agriculture (CGIAR, Cali, Colombia), were used in this study. Seeds were germinated in the dark, on water-soaked paper in Petri dishes, at 27 °C for two days, followed by three days at 23 °C. Seedlings were transferred to watered vermiculite trays and maintained in a growth chamber under the following conditions: 50% to 60% humidity, photoperiod of 12/12 h, day/night at 25/16 °C, respectively, and average light intensity of 400 µmol m^−2^ s^−1^. One week later, seedlings were transferred to 2.5 L pots with a (3:1) mixture standard “terra de Montemor” commercial soil (Horto do Campo Grande, Lisboa, Portugal) and vermiculite, respectively. Potted plants were maintained in growth chambers under the previously described conditions and watered three times per week during the whole assay. Flowers were tagged at anthesis, when the flower opens, and seeds were harvested at 10, 20, 30, and 40 days after anthesis (DAA) as described in [21]. Harvested seeds were immediately frozen in liquid nitrogen and stored at ‒80 °C until further use.

### 2.2. RNA Extraction, Quantification, and Quality Assessment

For total RNA isolation, frozen seeds were ground to a fine powder in liquid nitrogen using a mortar and pestle. An optimized version of the method of Chang et al. (1993) [22] was used for RNA isolation. Hexadecyltrimethylammonium bromide (CTAB), Ethylenediaminetetraacetic acid disodium salt dihydrate (EDTA), chloroform and sodium chloride (NaCl) were purchased from Merck (Darmstadt, Germany); Polyvinylpyrrolidone (PVP), β-mercaptoethanol and spermidine from Sigma-Aldrich (St. Louis, MO, USA), Tris-HCl from Carl Roth (Karlsruhe, Germany); isoamyl alcohol and lithium chloride from BDH Prolabo (Llinars del Vallès, Spain). 

Briefly, 900 µL RNA Extraction Buffer [(2% (w/v) CTAB, 2% (w/v) PVP, 100 mM Tris-HCl pH 8, 30 mM EDTA, 2 M NaCl, 0.5g/L spermidine and 3% β-mercaptoethanol (v/v))] was added to about 0.1 g of ground sample and incubated for 30 min at 65 °C. Two consecutive extractions with chloroform:isoamyl alcohol (24:1) were performed. In order to precipitate RNA, a LiCl solution was added to the supernatant to a final concentration of 3 M and incubated on ice for 30 min. RNA was recovered by centrifugation at 20,238 rcf for 20 min at room temperature. Two washes of the RNA pellets were performed with 70% and 100% absolute ethanol at ‒20 °C. Pellets were left to air-dry and diluted in 40 μL of cold Milli-Q RNAse-free water. Trace amounts of DNA contamination were removed with an Ambion^®^ TURBO™ DNase (Life Technologies, Carlsbad, CA, USA) following the manufacturer´s instructions. RNA quantification was performed using a NanoDrop^tm^ 2000c Spectrophotometer (Thermo Fisher Scientific Inc., Waltham, MA, USA). Extracted RNA purity was estimated based on the A260/280 and A260/230 ratios absorbance ratios and was approximately 2 before DNAse treatment. RNA integrity was assessed by electrophoresis in a 2.0% agarose gel, stained with SYBR^®^ Safe (Life Technologies). The absence of DNA contamination was verified by a standard polymerase chain reaction (PCR) using primers for the *P. vulgaris DEHYDRIN* mRNA, complete cds (gi|1326160) Forward 5′- AAGGAGAGGCGAAGGAGAAG -3′; and Reverse 5′- ACCAAACCCCACAACACAAC -3′. Samples were stored at ‒80 °C until needed.

### 2.3. Massive Analysis of 3’-cDNA Ends and Data Analysis

Massive analysis of 3’-cDNA ends (MACE) libraries, each representing one of the four time points corresponding to 10, 20, 30, and 40 DAA, were prepared using an equimolar pool of RNA from four biological replicates per time point. Each biological replicate correspond to seeds harvested from an individual plant. MACE libraries were prepared and sequenced by GenXPro GmbH (Frankfurt am Main, Germany) following in-house developed protocols [23,24]. Briefly, poly-adenylated mRNA was isolated from 1 μg of the large fraction of total RNA using Dynabeads^®^ mRNA Purification Kit (Life Technologies GmbH Invitrogen, Life Technologies). First- and second-strand synthesis of cDNA was performed using SuperScript^®^ III First-Strand Synthesis System (LifeTechnologies GmbH), with modified bar-coded 5′-end biotinylated poly-T adapters suitable for the Illumina Hiseq2000 flow cell (Illumina, San Diego, CA, USA). Subsequently, the cDNA was fragmented to yield 250 base pair (bp) fragments. The 3′-ends of the fragmented cDNA were captured with streptavidin beads, while PCR bias-proof technology ‘TrueQuant’ was used by ligation of TrueQuant adapters (GenXPro GmbH) to distinguish PCR copies from original copies [25,26]. The barcoded samples were sequenced simultaneously in one lane of an Illumina Hiseq2000 with 1 × 100 bps.

Analysis of MACE libraries started by removing low-quality sequence-bases using “cutadapt” (https://github.com/marcelm/cutadapt/) [27]. Poly(A)-tails were clipped by an in-house Python-Script. The reads were aligned to ‘Pvulgaris_442_v2.0.fa.gz’ (from Phytozome v12.0 *P. vulgaris*) using Bowtie 2 [28]. This tool maps reads depending on certain parameters (i.e., quality) and calculates thresholds for each sequence. The annotation information was taken from the files “Pvulgaris_442_v2.1.gene.gff3” and “Pvulgaris_442_v2.1.annotation_info.txt” (*Phaseolus vulgaris* v2.1, U.S. Department of Energy Joint Genome Institute, Phytozome v12.0: http://phytozome.jgi.doe.gov/). 

Normalization was performed using DEGseq R-package version 1.16.0 [29]. Genes were considered expressed (EGs) when they present a raw read value number >50 in at least one library. A semi-quantitative analysis of the changes in EGs was performed calculating the fold changes between two consecutive time points. (10 DAA vs. 20 DAA, 20 DAA vs. 30 DAA, 30 DAA vs. 40 DAA). All raw sequencing data have been deposited in NCBI Sequence Read Archive (SRA) database with the SRA accessions: SRR6466368, SRR6466367, SRR6466366 and SRR6466365. 

### 2.4. Primers and Probe Design 

Primers/probes for eight candidate genes selected from the DDR pathway were constructed: *SUPPRESSOR OF GAMMA RESPONSE 1* (*SOG1*; Phvul.003G027100), *ATAXIA-TELANGIECTASIA MUTATED* (*ATM*; Phvul.006G003000), *GAMMA HISTONE VARIANT H2AX* (*G-H2AX*; Phvul.006G095800), *DNA REPAIR AND MEIOSIS PROTEIN* (*MRE11*) (*MRE11*; Phvul.005G085700), *DNA REPAIR-RECOMBINATION PROTEIN* (*RAD50*; Phvul.001G266800), *NIJMEGEN BREAKAGE SYNDROME 1* (*NBS1*; Phvul.008G242800), *WEE1 KINASE HOMOLOG* (*WEE1*; Phvul.001G204900), and *NUCLEOSOME ASSEMBLY PROTEIN 1;2* (*NAP1;2*; Phvul.003G135500) (Table 1). Primers and TaqMan^®^ probes sequences were designed following the Primer Express^®^ Software Version 3.0 (Applied Biosystems, Foster City, CA, USA) guidelines and using Primer-BLAST (https://www.ncbi.nlm.nih.gov/tools/primer-blast). Primers and TaqMan^®^ probes were synthesized by Life Technologies. Conserved domain sequences were avoided to primer design in order to increase the specificity. Whenever possible, primer selection parameters were: primer size range of 20–26 bp, amplification product size range of 50–150 bp; primer melting temperature of 58–60 °C; primer GC content of 30–60%; primer with no more than two G/C in the last five 3’-end nucleotides and no more than three G nucleotides runs within the sequences. TaqMan^®^ probes design followed the same criteria, except size between 18 and 30 bp and melting temperature 68–70 °C. Probes were labeled with FAM™ or VIC^®^ dye on the 5’-end and Non-Fluorescent Quencher (NFQ) on the 3’-end (Table 1 and Table S1).

### 2.5. cDNA Synthesis and QuantStudio™ 3D Digital PCR

Four biological replicates per time point were used for the digital PCR (dPCR) assay. Reverse transcription was performed on the same RNA samples used for MACE sequencing. Briefly, 600‒900 ng of RNA was reverse-transcribed using oligo dT18 primers and the Promega ImProm-II^tm^ Reverse Transcription System (Promega, Madison, WI, USA) according to the manufacturer´s instructions. Resulting cDNAs were diluted to a final concentration of 10 ng/μL.

After assessing efficiency and specificity, dPCR was used to quantify the candidate gene expression. QuantStudio™ 3D Digital PCR 20K Chip Kit v2 (Thermo Fisher Scientific Inc.) was used to perform the dPCR according to the manufacturer’s instructions. The standard cycling protocol was run on a GeneAmp PCR system 9700 thermal cycler (Thermo Fisher Scientific Inc.): 10 min at 96 °C, 40 cycles of 2 min at 60 °C and 30 s at 98 °C, final extension step of 2 min at 60 °C. QuantStudio™ 3D Digital PCR Instrument was used to process the chips, by capturing the image and performing a quantitative analysis of the target DNA concentration by interpolation of the fraction of positive reactions, labeled by FAM and VIC probes, with a Poisson distribution [30]. Data were then transferred to the Thermo Fisher Cloud where the QuantStudio™ 3D Analysis Suite™ Software further evaluated the chip quality. The confidence level was set at 95%, desired precision at 10%, in the Poisson Plus algorithm version 4.4.10. The theoretical confidence interval for each probe and time point was calculated by multiplying the obtained copies/µL (*n* = 4) by the corresponding precision value.

### 2.6. Bioinformatic Analysis

Functional characterization was performed using the MapMan web tools (http://www.plabipd.de/portal/mercator-sequence-annotation) [31,32,33]. Unspliced gene sequences of all expressed genes were obtained using BioMart in Phytozome v.12 (https://phytozome.jgi.doe.gov/) to create a mapping file for the Mercator pipeline. A BLAST analysis was conducted on histone superfamily protein gene sequences via BLASTN (https://blast.ncbi.nlm.nih.gov/) using megablast algorithm, against the Nucleotide collection (nr/nt) database, in Viridiplantae.

Cytoscape [34] software (Version 3.6.1) was used to visualize the molecular interaction networks. To ease the analysis, molecular interaction networks were established using the EGs from MapMan “DNA” category between two consecutive time points (10 DAA vs. 20 DAA, 20 DAA vs. 30 DAA and 30 DAA vs. 40 DAA) with a two-fold change in expression levels. For the same EGs, complementary functional categories resulting from MapMan categorization were retrieved. The inputs for the network analyses were the MapMan BinName as the target node and the Gene ID as the source node.

A comparison between DNA-related genes and their respective proteins found in Parreira et al., 2016 [21] was established. Proteins differentially accumulated were identified from the seed samples collected in an independent experiment at the same time points as the ones studied herein. The expression profiles were established using the Log_2_ of the average normalized intensity values for protein abundance, and the Log_2_ of the normalized expression values for the genes.

## 3. Results

### 3.1. Global Overview of the Gene Expression during Seed Development

Four MACE libraries (10, 20, 30, and 40 DAA) were prepared to capture the time frame of transcriptome changes underlying major SD stages. Seed samples harvested at 10 DAA represent the late embryogenic stage, in which evidence of a high rate of cell division associated with embryo differentiation and morphogenesis has been described by us in a previous study [21]. At 20 DAA seeds are at the maturation/filling stage and an increased biomass accumulation is seen as a result of the synthesis of storage reserves. The 30 DAA represents the end of the filling stage marked by the end of biomass accumulation and the beginning of seed dehydration up until 40 DAA, when seeds are desiccated. 

MACE sequencing of the four cDNA libraries resulted in 41.6 million 94-bp reads, with an average of 10.4 million reads/sample (Table 2).

A total of 14,001 genes were expressed and identified among all the samples analyzed in this study (Table S3), representing 51.04% of the total number of loci described in the *Phaseolus vulgaris* v2.1 genome available at Phytozome v12.0 [35]. Sequences identified as *LOW-MOLECULAR-WEIGHT CYSTEINE-RICH 68* (*LCR68*; Phvul.005G071300), *LECTIN RECEPTOR KINASE A4.3* (*LECRKA4.3*; Phvul.004G158100) and *CUPIN FAMILY PROTEIN* (*PAP85*; Phvul.007G059775) were amongst those with highest total raw read counts, suggesting no ribosomal RNA (rRNA) contamination during library preparation.

MapMan functional categorization identified the major biological and metabolic processes occurring during SD. “Protein” [BinCode (BC) 29] is the most representative category with 14.80% of assigned EGs, followed by “RNA” (BC 27; 10.65%), “signaling” (BC 30; 5.58%), “miscellaneous” (BC 26; 4.61%), “transport” (BC 34; 3.64%), “cell” (BC 31; 3.53%) and “development” (BC 33; 2.70%) (Table S2). The functional category “not assigned” accounts for 34.09% of the EGs. 

### 3.2. Genes Involved in DNA Damage Response and Chromatin Remodeling during Seed Development

**Figure 1:**
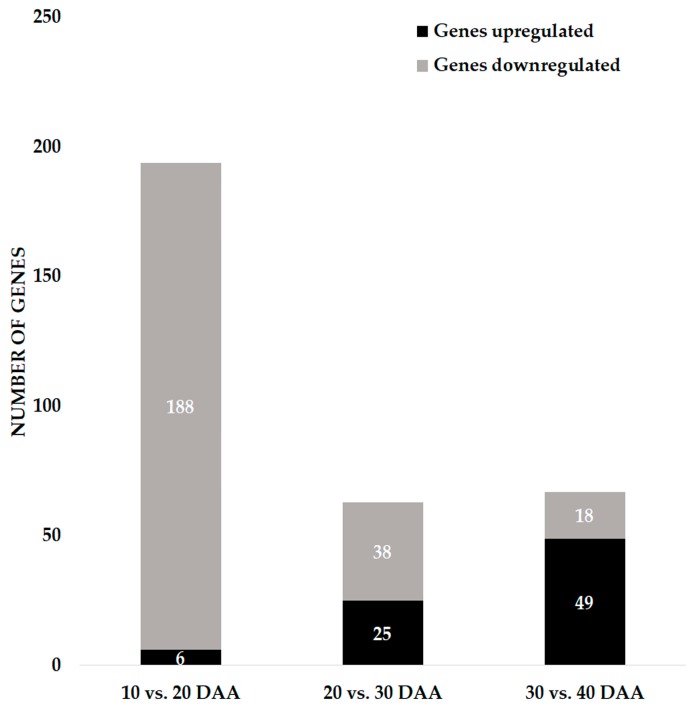
The number of up- and downregulated expressed genes belonging to DNA functional category in developing seeds of *P. vulgaris* with a minimum of two-fold change in the expression. DAA: days after anthesis.

Genes were retrieved from MACE datasets associated with DDR (sensing, signal transduction, and repair) and chromatin remodeling, expressed during the time frame of SD. To accomplish this goal, we started to investigate EGs belonging to the MapMan functional category of “DNA” (BC 28) (Table S4). Among the 301 EGs identified in the “DNA” category, 67.11% (202 EGs) were assigned to the DNA.synthesis/chromatin structure (BC 28.1) sub-category, 15.61% (47 EGs) were assigned to DNA repair (BC 28.2), and 20.60% (62 EGs) were assigned to DNA.unspecified (BC 28.99). A semi-quantitative analysis of the changes in the expression of these genes was performed, calculating the fold changes between two consecutive time points. Among the 301 EGs identified, 194 showed fold changes higher than 2 between 10 DAA and 20 DAA, 63 between 20 DAA and 30 DAA, and 67 between 30 DAA and 40 DAA (Figure 1). In the transition between 10 DAA to 20 DAA, 96.9% of the “DNA” category genes were found to be downregulated. On the other hand, on the transition between 30 DAA and 40 DAA the expression of 73.13% of EGs was upregulated.

**Figure 2:**
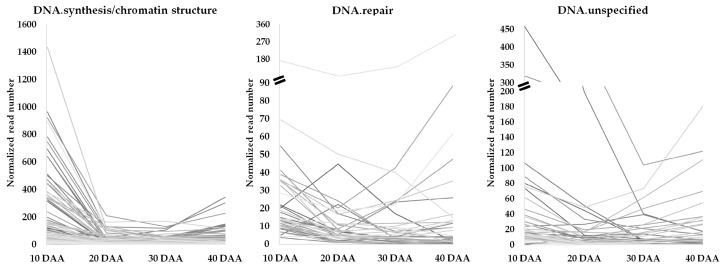
Profiles of expressed genes classified in the MapMan functional “DNA” subcategories during *P. vulgaris* seed development. Grey lines depict expression profiles for each individual gene in the subcategory with at least two-fold change in the expression.

For each sub-category, gene expression profiles for EGs were established (Figure 2). The EGs belonging to the “DNA.repair” subcategory show relatively lower expression when compared to the normalized read values observed in “DNA.synthesis/chromatin structure”.

Forty-four EGs encoding histones were identified, representing 21.8% of EGs included in the subcategory “DNA.synthesis/chromatin structure”. The *GAMMA VARIANT OF HISTONE H2AX* (*G-H2AX*; Phvul.006G095800) is highly expressed at 10 DAA, decreasing strongly its expression levels in the next stage studied (Table S4). Increase in the expression of some members of the *HISTONE SUPERFAMILY PROTEIN* were noticed on the transition from 30 DAA to 40 DAA. The BLAST analysis conducted on histone superfamily protein gene sequences revealed that the majority has strong homology with predicted members of H3 (H3.2) and H4 family (see Table S5 for more information).

A decrease in expression from 10 to 20 DAA was observed for the majority of genes categorized in this “DNA” subcategory (Figure 2, such as the *NUCLEOSOME ASSEMBLY PROTEIN 1;2* (*NAP1;2*; Phvul.009G231400), the *SPO11/DNA TOPOISOMERASE VI, SUBUNIT A PROTEIN* (*BIN5*; Phvul.007G162700) the *RPA70-KDA SUBUNIT B* (*RPA70B*; Phvul.003G184400) and the *TOPOISOMERASE II* (*TOPII*; Phvul.005G024200). The expression of *MCM2*, *MCM3*, *MCM4*, *MCM5*, *MCM6*, *MCM7* and *MCM9 MINICHROMOSOME MAINTENANCE (MCM2/3/5) FAMILY PROTEIN* (Phvul.003G16100, Phvul.002G194200, Phvul.011G041300, Phvul.003G175800, Phvul.009G245600, Phvul.001G212600 and Phvul.006G204900, respectively), related with DNA replication [36], show also high expression at 10 DAA decreasing afterwards. The *CHROMATIN REMODELING FACTOR17* (*CHR17*; Phvul.003G101700) and the *ORIGIN RECOGNITION COMPLEX 1* (*ORC1A*; Phvul.L009243) are also highly expressed at 10 DAA but the expression also decreases strongly at 20 DAA. Nevertheless, some exceptions to this trend were noticed. That is the case of the *RESTRICTION ENDONUCLEASE TYPE II-LIKE SUPERFAMILY PROTEIN (RAD1*; Phvul.002G001900), which increases strongly between 20 and 30 DAA and, the *SMR (SMALL MUTS RELATED) DOMAIN-CONTAINING PROTEIN* (Phvul.006G068100), which increases from 30 to 40 DAA (Table S4). 

Within the “DNA.repair” subcategory, the *MUTL PROTEIN HOMOLOG 3* (*MLH3*; Phvul.002G116900) expression decreases from 10 to 20 DAA and increases afterward between 20 and 30 DAA (Table S4). On the contrary, the *MUTS HOMOLOG 2* (*MSH2*; Phvul.003G177100), *MUTS HOMOLOG 6* (*MSH6*; Phvul.001G212500) and *MUTS HOMOLOG 7* (*MSH7*; Phvul.004G162000) decrease in abundance from 10 to 20 DAA (Table S4). The *LEUCINE-RICH REPEAT (LRR) FAMILY PROTEIN* (*DNA-DAMAGE-REPAIR/TOLERATION PROTEIN 100 - DRT100*; Phvul.011G212100) has the highest expression observed at 20 DAA, decreasing afterward. The *DNA-DAMAGE-REPAIR/TOLERATION PROTEIN* (*DRT102*; Phvul.006G099800) also shows a peak in expression at 20 DAA, decreasing afterwards, namely on the 30 to 40 DAA. Still, in the “DNA.repair” subcategory, the *REPLICON PROTEIN A2* (*RPA2*; Phvul.003G145200) and *RAS ASSOCIATED WITH DIABETES PROTEIN 51* (*RAD51*; Phvul.003G126800) showed decreased expression from 10 to 20 DAA. Three *DNA GLYCOSYLASE SUPERFAMILY PROTEINS* (Phvul.003G156200, Phvul.005G045900 and Phvul.003G197200) have a high expression at 10 DAA, decreasing at 20. Interestingly, part of the MRN complex, the *DNA REPAIR AND MEIOSIS PROTEIN* (*MRE11*; Phvul.005G085700) and the *DNA REPAIR-RECOMBINATION PROTEIN* (*RAD50*; Phvul.001G266800), show high expression at 10 DAA, decreasing to 20 DAA (Table S4).

Under the “DNA.unspecified” subcategory, the *CHROMATIN REMODELING FACTOR CHD3 (PICKLE)* (*PKL*; Phvul.001G046100) shows a strong decrease in its expression from 10 to 20 DAA (Table S4). The WHIRLY 2 (WHY2; Phvul.006G106800) was also highly expressed at 10 DAA (Table S4).

Other genes not categorized as “DNA” and implicated in DNA damage response mechanisms were also retrieved from our MACE datasets (Table S3). As an example, we found that the expression of the *HOMOLOG OF DNA MISMATCH REPAIR PROTEIN MSH3* (Phvul.007G069100), categorized as “Signalling.G-proteins,” increases from 30 to 40 DAA. Expression of *RELATED TO UBIQUITIN 1* (*RUB1*; Phvul.005G060100), categorized as “Protein.degradation,” decreases from 10 to 20 DAA. The expression of the *SUPPRESSOR OF GAMMA RESPONSE 1* (*SOG1*, Phvul.003G027100), categorized as “Development.unspecified,” shows a strong decrease from 10 to 20 DAA, increasing afterwards. The expression of *ATM* (Phvul.006G003000) and *WEE1* (Phvul.001G204900), two kinases implicated in transduction of DNA damage that belong to the MapMan “Protein.postranslational modification” category, showed strong decrease in their expression in the transition from 10 DAA to 20 DAA.

**Figure 3:**
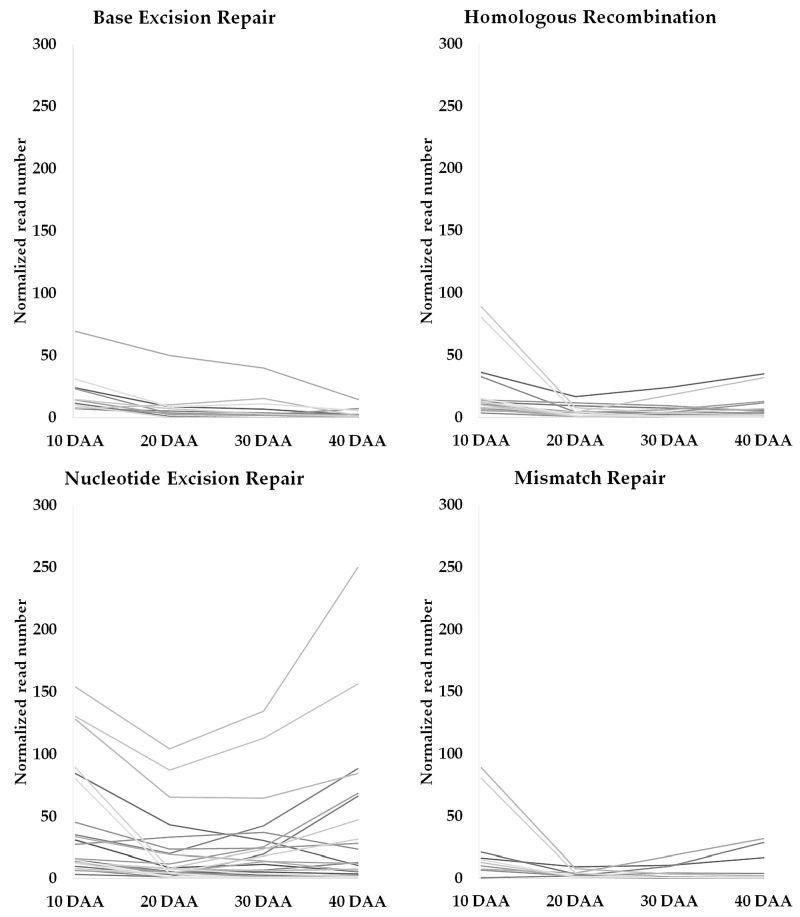
Profiles of expressed genes related with different DNA repair mechanisms (classification based on description made by Sampinato [4]). DAA: days after anthesis. Grey lines depict expression profiles for each individual gene in the DNA repair mechanisms.

We also retrieved a collection of EGs related to base excision repair (BER), nucleotide excision repair (NER), homologous recombination (HR), mismatch repair (MMR), and non-homologous end joining (NHEJ) based on the categorization done by [4] (Table S6). The analysis of this data shows a higher number of NER-related genes was found when compared with the number of genes from other repair mechanisms throughout SD (Table S6). The expression profiles of these genes were established (Figure 3) and, importantly, a trend showing an increase of gene expression on the transition from 30 DAA to 40 DAA was observed on genes related to NER.

### 3.3. Quantification by QuantStudio™ 3D Digital PCR

**Figure 4:**
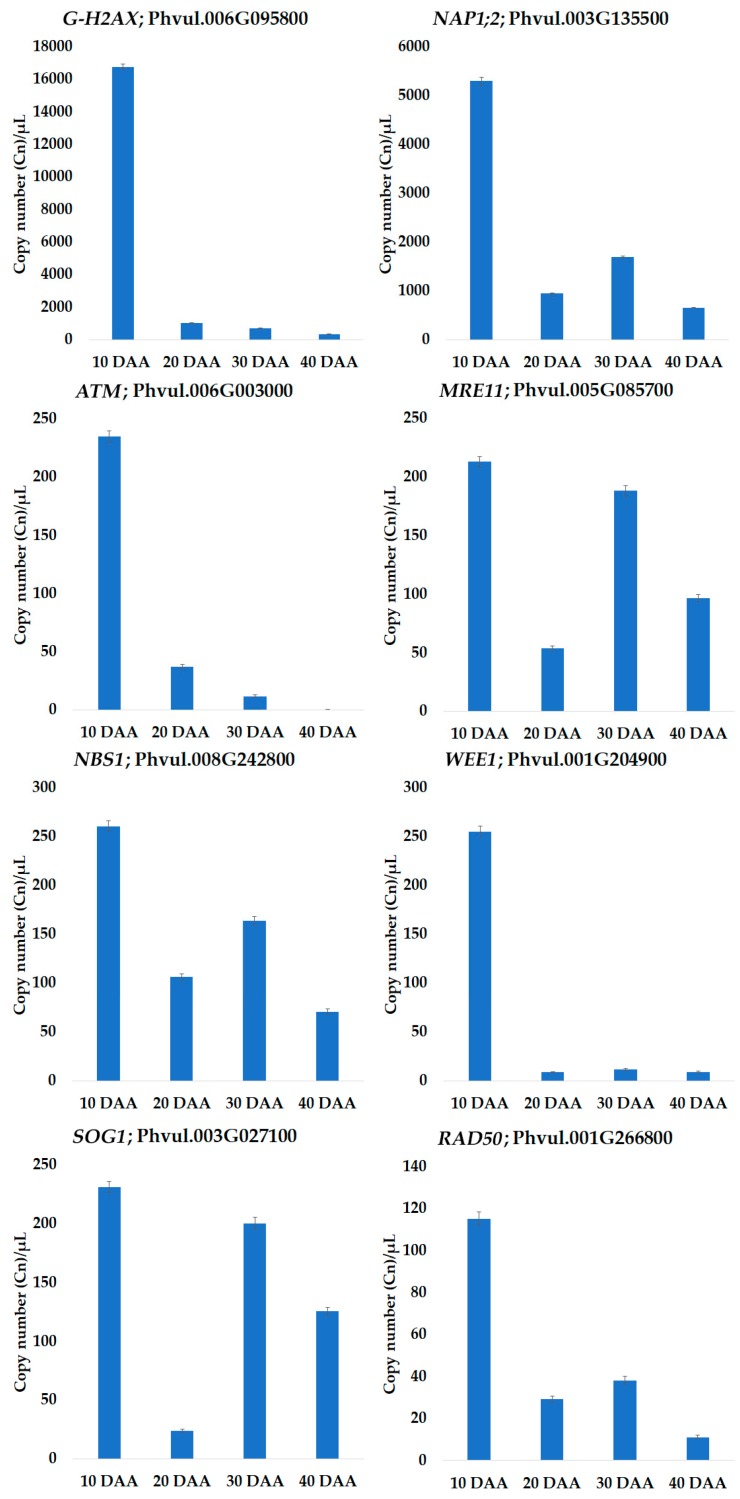
Bar plots represent mRNA copy number per microliter (Cn/µL) of biological quadruplicates for the eight genes selected for digital PCR. The Cn/µL was calculated using QuantStudio™ 3D Analysis Suite™ assuming a Poisson distribution; error bars correspond to the theoretical confidence interval. The precision of quantification of *ATM* at 40 DAA and *WEE1* at 20 and 40 DAA was higher than 10%. Y-axis: Cn/µL; X-axis: days after anthesis (DAA).

The MACE study provided evidence that the expression of genes implicated in DDR and chromatin remodeling occurs at relatively low levels and changes during seed development. To corroborate these assumptions, the expression levels of eight genes involved in DNA damage sensing were quantified by dPCR in four biological replicates. The transcript copy number per microliter (Cn/µL) variation of the eight genes studied during the SD is shown in Figure 4.

For all tested genes, the highest Cn/µL, was observed at 10 DAA. From 10 DAA to 20 DAA, all genes show a strong decrease in expression, being *WEE1* the most downregulated gene with a Log_2_ fold change (FC) ‒4.89. Interestingly, an upregulation of *SOG1* and *MRE11* at 30 DAA was noticed, showing a Log_2_FC 3.05 and 1.81, respectively. To a lesser extent, this was also observed for *NAP1;2*, *RAD50*, and *NBS1* (Figure 4).

A positive correlation (*R^2^* = 0.829) between the Log_2_ of the expression values obtained by the two approaches (dPCR and MACE) was established for the eight genes analyzed, using the data from the four studied time points (Figure S1). This high correlation seems to support the accuracy of the MACE analysis.

### 3.4. Changes in Transcriptomic Profiles Are in Accordance with Proteome Changes

**Figure 5:**
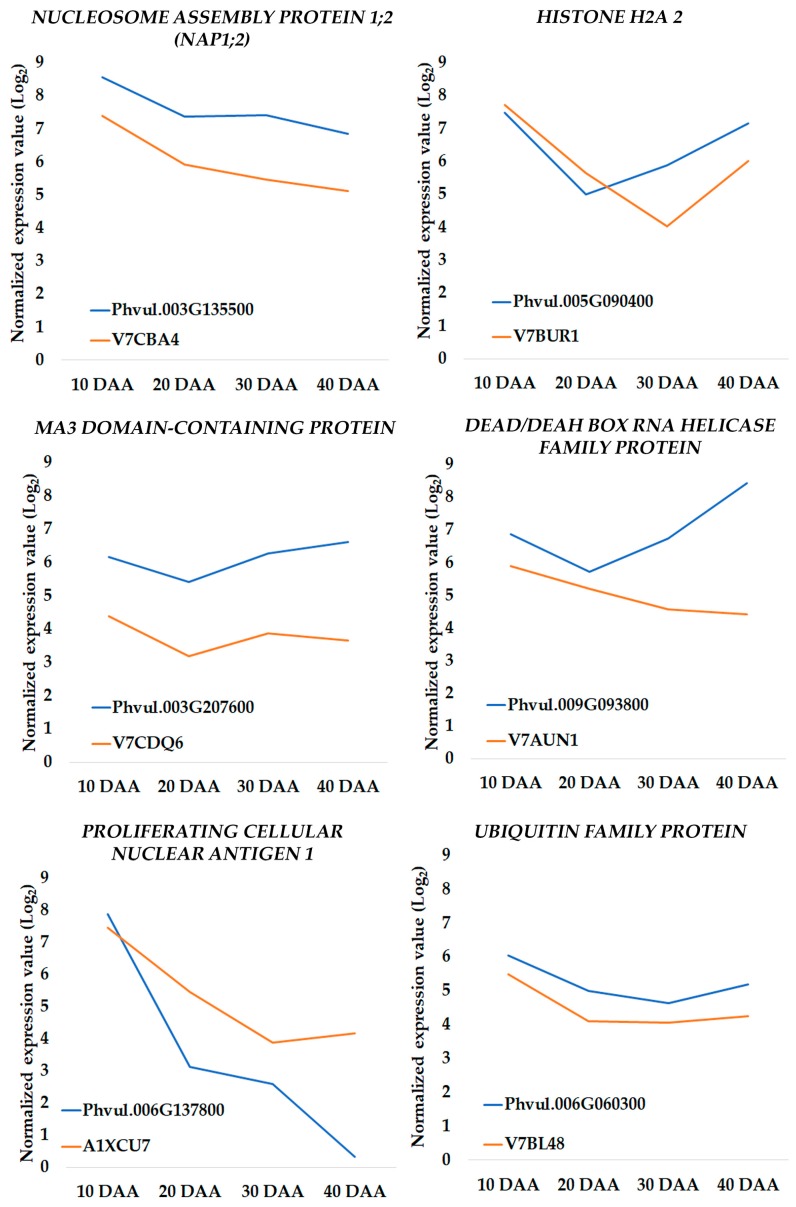
Abundance profiles of proteins [21], and expression of their correspondent genes, related to the DNA metabolism. Line in blue: Log_2_ of the normalized expression values of the gene. Line in orange: Log_2_ of the average normalized intensity values of the protein.

To increase the robustness of our study, we investigated whether the changes detected in the transcriptome were in agreement with the changes observed in the proteome, using data from a previous study [21]. Only six proteins related with DNA metabolism were identified and their accumulation profiles were compared with the expression profiles of their protein-coding genes, *HISTONE H2A 2* (Phvul.005G090400; V7BUR1), *NUCLEOSOME ASSEMBLY PROTEIN 1;2* (*NAP1;2*) (Phvul.003G135500; V7CBA4), *MA3 DOMAIN-CONTAINING PROTEIN* (Phvul.003G207600; V7CDQ6), *DEAD/DEAH BOX RNA HELICASE FAMILY PROTEIN* (Phvul.009G093800; V7AUN1), *PROLIFERATING CELLULAR NUCLEAR ANTIGEN 1* (Phvul.006G137800; A1XCU7), and *UBIQUITIN FAMILY PROTEIN* (Phvul.006G060300; V7BL48). As observed in Figure 5 there is a reasonable agreement between the transcriptomic and proteomic profiles obtained in the two independent experiments.

### 3.5. Network Analysis

Network analysis was conducted using EGs belonging to the “DNA” MapMan category that presented at least 2-fold changes between consecutive seed development stages. This analysis provides an integrative visualization of transcriptomic datasets allowing the identification of EGs that have more than one MapMan functional annotation besides “DNA”, thus evidencing additional functions. 

#### 3.5.1. Network Analysis from 10 to 20 Days after Anthesis

**Figure 6:**
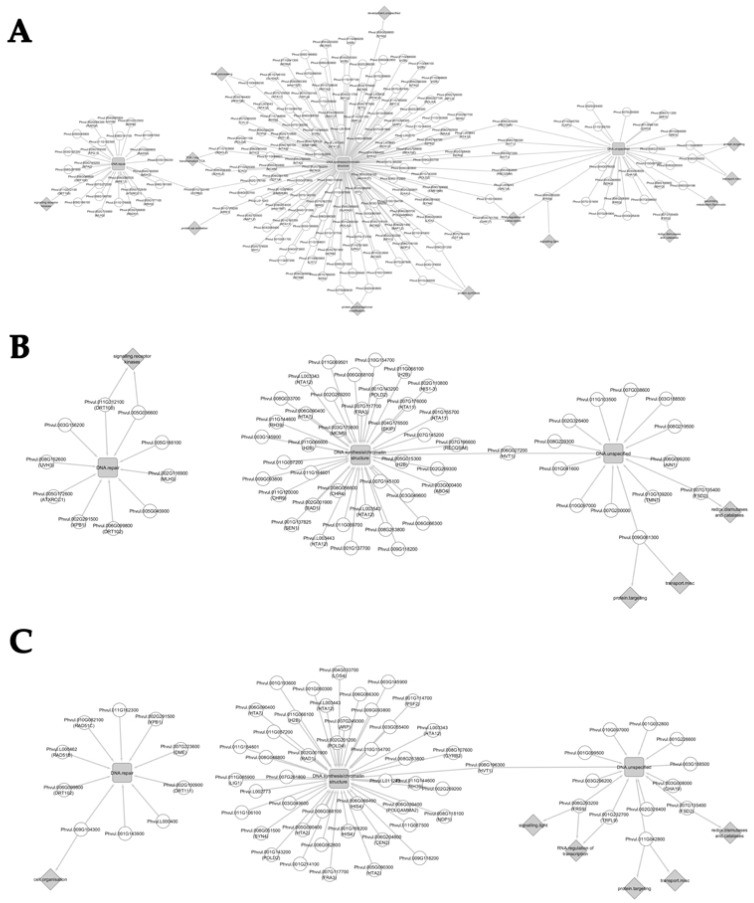
Network analysis of expressed genes between two consecutive seed development stages using the expressed genes (EGs) belonging to MapMan functional category “DNA” that changed expression at least 2-fold. For the same EGs, complementary functional categories resulting from MapMan categorization are also evidenced. Grey squares represent MapMan functional subcategories of “DNA”, gray diamonds represent other MapMan functional categories beside “DNA” and white circles represent EGs with changed expression in a comparison. Comparisons between time points are shown: (**A**) 10 DAA vs. 20 DAA; (**B**) 20 DAA vs. 30 DAA; (**C**) 30 DAA vs. 40 DAA. DAA: days after anthesis.

Network analysis was conducted with the 194 EGs between 10 DAA and 20 DAA. Several connections between “DNA” functional category and other functional categories such as “RNA.regulation of transcription", "TCA/org transformation.TCA”, “protein.synthesis” were unveiled, reflecting possible interactions between different metabolic pathways (Figure 6A). 

The previously described *ORC1A* (Phvul.L009243), involved in DNA replication [37], and the *HELICASE IN VASCULAR TISSUE AND TAPETUM* (*HVT1*; Phvul.008G196300 and Phvul.006G027200) bridges “DNA.unspecified” with “DNA.synthesis/chromatin structure” subcategory. Other DNA helicases as *RECQ4A* (Phvul.006G216200) and *RECQ HELICASE SIM* (*RECQSIM*; Phvul.006G082600), involved in maintenance of genome integrity [38,39], also connect these two above mentioned functional subcategories. The *DNA GYRASE B2* (*GYRB2*; Phvul.001G123100), which plays a role in the control of DNA topology [40], also bridges “DNA.synthesis/chromatin structure” to “DNA.repair”.

Other functional categories were found connected with the “DNA” categories and sub-categories. As an example, the *SEC14P-LIKE PHOSPHATIDYLINOSITOL TRANSFER FAMILY PROTEINS* (Phvul.011G042800 and Phvul.009G006000), related with membrane trafficking [41], is connecting “DNA.unspecified” to “protein.targeting” (BC 29.3) and to “transport.misc” (BC 34.99). The “redox.dismutases and catalases” (BC 21.6) is connected to “DNA.unspecified” via the *FE SUPEROXIDE DISMUTASE 2* (*FSD2*; Phvul.007G135400). 

Among other different connections established, we noticed that the *FAR1-RELATED SEQUENCE 9* (*FRS9*; Phvul.008G293200), a putative negative regulator specific to phyB signaling [42], bridges “DNA.unspecified” to “signalling.light” (BC 30.11) and “RNA.regulation of transcription” (BC 27.3). On the other side, the *CHROMATIN REMODELING FACTOR17* (*CHR17*; Phvul.003G101700) also bridges “RNA.regulation of transcription” with “DNA.synthesis/chromatin structure”. *RPA70B*, previously described in Section 3.2 bridges “RNA.processing” (BC 27.1) with “DNA.synthesis/chromatin structure”. The *RAD21/REC8-LIKE FAMILY PROTEIN* (*SYN3*; Phvul.005G038800) is an essential gene for megagametogenesis [43] links “development.unspecified” (BC 33.99) to “DNA.synthesis/chromatin structure” in our networks. Interestingly, EGs categorized as “DNA.synthesis/chromatin structure” (e.g., *HISTIDYL-TRNA SYNTHETASE 1*, Phvul.001G179200; *KINASE INTERACTING (KIP1-LIKE) FAMILY PROTEIN*, Phvul.007G060600 and Phvul.002G043800) were also categorised as “protein” subcategories (BC 29.1; BC 29.4). “Signalling.receptor kinases” (BC 30.2) and “DNA.repair” is bridged by *DRT100*, already described in Section 3.2. 

#### 3.5.2. Network Analysis from 20 to 30 Days after Anthesis

The network analysis of 20 to 30 DAA, constructed with 63 EGs, revealed that the subcategory of “DNA.repair” is no longer connected to “DNA.synthesis/chromatin structure” (Figure 6B). Nonetheless, the *HVT1* (Phvul.006G027200) bridges “DNA.unspecified” with “DNA.synthesis/chromatin structure” as observed for the 10 to 20 DAA comparison.

The *FE SUPEROXIDE DISMUTASE 2* (*FSD2*; Phvul.007G135400), involved in ROS scavenging [44] bridges the “redox.dismutases and catalases” (BC 21.6) to “DNA.unspecified”. The *SEC14P-LIKE PHOSPHATIDYLINOSITOL TRANSFER FAMILY PROTEIN* (Phvul.009G061300) bridges “protein.targeting” (BC 29.3) and “transport.misc” (BC 34.99) with “DNA.unspecified”. Also, the *LEUCINE-RICH REPEAT (LRR) FAMILY PROTEIN* (Phvul.005G036600) and the *DRT100* (Phvul.011G212100) are bridging “signalling.receptor kinases” (BC 30.2) with “DNA.repair”.

#### 3.5.3. Network Analysis from 30 to 40 Days after Anthesis

The last network established with the 67 EGs of the “DNA” functional category also revealed no connection between the “DNA.synthesis/chromatin structure” and the “DNA.repair” (Figure 6C). As seen in a previous network (Figure 6A), the *HVT1* (Phvul.008G196300) bridges “DNA.unspecified” with “DNA.synthesis/chromatin structure”. The *FSD2* (Phvul.007G135400) bridges “redox.dismutases and catalases” (BC 21.6) with “DNA.unspecified”, while the *SEC14P-LIKE PHOSPHATIDYLINOSITOL TRANSFER FAMILY PROTEIN* (Phvul.011G042800) bridges “protein.targeting” (BC 29.3) and “transport.misc” (BC 34.99) with “DNA.unspecified”. Among other connections established, the *TRF-LIKE 9* (*TRFL9*; Phvul.001G232700) that encodes for a telomeric DNA-binding protein [45] bridges “DNA.unspecified” with “RNA.regulation of transcription” (BC 27.3). Interestingly, the *FRS9* (Phvul.008G293200) bridges “DNA.unspecified” with “RNA.regulation of transcription” (BC 27.3) and “signalling.light” (BC 30.11). 

## 4. Discussion

We characterized the transcriptomic landscape of *P. vulgaris* seeds at the stages of late embryogenesis (10 DAA), early (20 DAA) and late filling (30 DAA), and seed desiccation (40 DAA). While the maintenance of genome integrity has been thoroughly investigated in seed germination [12,14,15] or in seed response to priming agents [46], information is still scarce in relation to seed development.

Despite the sequencing of a single pooled MACE library for each time point analyzed, the identification of expressed genes implicated in DNA damage response (DDR) and chromatin remodeling in the time frame of seed development was possible. Our MACE approach did not allow the assessment of biological variance contribution [47], but still provided new insights into the mechanisms that seeds likely use to cope with DNA damage to maintain genome integrity and seed viability upon desiccation when grown under optimal growth conditions. Still, we cannot disregard that, when the plant matures under field conditions, with a certain level of environmental disturbance (e.g., abiotic stresses), different molecular responses may occur at the seed level. More studies would be needed to elucidate these aspects.

Among the different DDR components found expressed during seed development, we focused our attention on those acting upstream DNA repair, like the DSB sensing and signal transduction components (Figure S2). Digital PCR (dPCR) allows absolute transcript quantification, even at low expression levels, due to its improved sensitivity [48,49] and precision, especially in low-concentration samples [48,50,51,52]. In this work we have used a chip-based platform, the QuantStudio 3D Digital PCR to quantify expression levels of eight genes involved in DSB sensing and signal transduction. This methodology was previously successfully applied to plants [53]. Although different technologies can report different expression levels for the same gene [47], our dPCR results showed a high positive correlation with the MACE ones (Figure S1). Moreover, a similarity between the transcriptomic and proteomics profiles was obtained (Figure 5), when gene expression data are compared with proteomic data from an independent experiment carried with four biological replicates for the same *P. vulgaris* seed development time points [21]. This supports the accuracy of the information provided by the transcriptomic study hereby presented.

With the inherent limitations described previously, our MACE study highlights a qualitative timeframe of molecular events associated with the maintenance of genome integrity during seed development. DNA damage sensing and the different repair mechanisms seem to be activated during early SD stages, when cell division and differentiation occurs. Chromatin structure and nucleotide excision repair seem to be relevant during seed dehydration, evidencing the activation of seed protection mechanisms that could play a major role on seed viability. Additionally, the molecular interaction networks established evidence other functional categories putatively related to DNA metabolism that may act in a concerted way during SD. These results are discussed in the following sections.

### 4.1. Tight Control of DNA Damage Seems to Occur during Seed Development

A generic downregulation of genes belonging to the MapMan “DNA” category was observed in the transition between 10 to 20 DAA. The 10 DAA time point reflects a late embryogenesis stage, in which a high rate of cell division has been reported [21,54,55,56] and consequently an increased expression of DNA replication and repair factors is expected. Our results are in agreement with this. As stated previously, the detection of DNA double-strand breaks (DSB) is necessary to initiate DSB repair. Our MACE study provided us indications that the expression of DSB sensing and signal transduction components may change during SD and we corroborated this information by digital PCR. As one example, the protein kinase *ATAXIA-TELANGIECTASIA MUTATED* (*ATM*, Phvul.006G003000; Table S3), a DNA damage-inducible protein kinase that phosphorylates a plethora of substrates participating in DNA damage response [7], was found to be highly expressed at 10 DAA, decreasing afterward, as seen in our dPCR results (Figure 4). While Waterworth et al. (2016) reported the crucial role played by ATM in genome safeguarding during seed germination [1], our findings suggest a similar role for ATM during the first stages of seed development.

Besides ATM, the DSB sensing mechanism requires the complex interaction of several other DDR components. In *Arabidopsis*, the activation of ATM is dependent on a functional MRN (MRE11-RAD50-NBS1) complex [7,57], whereas ATM activates the phosphorylation of the DDR master regulator SUPPRESSOR OF GAMMA RESPONSE 1 (SOG1). The latter modulates the transcriptional response during DDR, triggering activation of cell cycle checkpoints and eventually programmed cell death [8,9,58]. Also, the *WEE1* kinase expression is regulated in an ATM-dependent manner, inhibiting the cell cycle upon activation of the DNA integrity checkpoint [59]. On the other side, the histone H2AX is also phosphorylated by ATM, in response to DNA damage, resulting in γH2AX foci, which are the sites for recruitment of DDR factors [3,57,60] (Figure S2). Chaperones, as the NAP1-RELATED PROTEIN 2, a member of NUCLEOSOME ASSEMBLY PROTEIN-1 (NAP1) family of histone H2A/H2B have been described as having a role in nucleosome assembly and histone trafficking during DNA repair [61]. As observed for *ATM*, relevant components of the DSB sensing mechanism, the MRN complex genes, *SOG1*, and *WEE1* have a high expression at 10 DAA in our dPCR results (Figure 4). Similar results were obtained for the *GAMMA HISTONE VARIANT H2AX* and *NAP1;2*. It is not surprising that a tight control of DNA integrity is needed, due to the high rate of cellular division and metabolic activity characteristic of this stage [21,55]. Indeed, even in the absence external stresses, DNA damage can arise from endogenous ROS, metabolic by-products, and breaks induced during DNA replication [14,15]. Another interesting aspect is the major upregulation of *SOG1*, *NAP1*, *MRE11*, *NBS1* and *RAD50* observed at 30 DAA in the dPCR profiling. From our previous study [21], we found that seed fresh weight decreased from 30 DAA, an aspect associated with the end of the seed growth and the onset of dehydration. Numerous reports highlights that the high metabolic activity in developing seed and drying of mature seed results in the production of ROS, such as superoxide radical (O^2−^) and hydrogen peroxide (H_2_O_2_) [62,63]. In *Helianthus annuus*, the H_2_O_2_ content is quite high at the beginning of seed development, probably because the moisture content is high enough to allow metabolic activities [62]. In this context, we could suggest that upregulation in DSB sensing components at 30 DAA maybe be a consequence of the production and accumulation of ROS capable of genotoxic damage. In *Medicago truncatula* seeds, we have seen that upregulation of DNA response components occurs, being these implicated in the maintenance of genome stability in response to the genotoxic stresses applied [12,64]. Although, the expression levels of the histone *H2AX* and *ATM* seems to decrease slightly from 20 DAA to 40 DAA, we cannot discard their role in DDR, since these components act at the post-translational level [7]. 

Other components of the response to DNA damage seem to be expressed during *P. vulgaris* seed development. One of these components is the PROLIFERATING CELLULAR NUCLEAR ANTIGEN (PCNA), a scaffold protein required to recruit DNA repair components at the damaged sites [65]. We found evidence that *PCNA1* (Phvul.006G137800) is highly expressed at 10 DAA, decreasing afterward during seed development. Although we did not validate the expression of this gene by dPCR, we noticed the same trend of accumulation for this protein (A1XCU7) based on the results of previous proteomic study conducted on similar seed samples [21] (Figure 5; Table S3). In humans, PCNA is also described as interacting with MUTS proteins [66], heterodimers composed of distinct MUTS homologs (MSH) subunits involved in mismatch repair mechanism (MMR) [4]. MutSα (MSH2/MSH6) and MutSβ (MSH2/MSH3) are eukaryotic mismatch recognition proteins that preferentially process base‒base and small insertion/deletion (ID) mispairs, respectively [67]. We have detected considerable expression of the *MSH2* and *MSH6* subunits at 10 DAA. The expression of the *MSH3* subunit increases remarkably in the transition from 30 DAA to 40 DAA, when the seed starts to dehydrate and cellular components are prone to oxidative damage. The presence of MUTS and PCNA homologs in plant tissues suggest that an interaction between these components may occur, as described with humans, but, to the best of our knowledge, this has not yet been described in plants and still deserves further validation.

The REPLICATION PROTEIN A (RPA), an essential regulator of eukaryotic DNA metabolism, has a key role in DDR, binding to single-stranded DNA (ssDNA) and coordinating the recruitment and exchange of genome maintenance factors [68]. RPA is involved in the sensing of lesions that cause stalled replication forks, as well as in NER, MMR and HR pathways [4,68,69]. Similar to what was observed for other DDR genes, and based on limited evidence from the MACE study, the expression of several RPA subunits, such as *RPA70B*, is high at 10 DAA and decreases at 20 DAA. 

One question that remains to be elucidated is whether the profile of expression of DDR genes found at late embryogenesis is similar to the one found in other non-embryogenic proliferating tissues such as the shoot apical meristem (SAM) and root apical meristem (RAM). In rice, Kimura et al. [70] compared the expression patterns of DDR genes in proliferative tissues (SAM and RAM) and non-proliferative tissues as mature leaves. Among others, *OsPCNA*, *OsRPA32*, and *OsORC1* were found to be expressed in both proliferating tissues but not mature leaves. Interestingly, our results show a high expression of *PCNA1*, *ORC1A* and *RPA* subunits at 10 DAA (Table S3), which suggests a similarity in DNA damage responses between tissues where cell division and differentiation are actively occurring. Nevertheless, more comprehensive studies are required to further validate this evidence.

Another aspect revealed by this study is the peak expression at 20 DAA for the *DNA-DAMAGE REPAIR/TOLERATION 100 PROTEIN* (*DRT100*), which matches the early filling and change in seed coat coloration. Our network analysis in the transition from 10 DAA to 20 DAA revealed that *DRT100* bridges “DNA.repair” and “signaling.receptor kinases” Mapman functional categories. DRT100 is a putative homolog of RecA chloroplastidial protein, a protein that plays important roles in DNA damage repair [71]. The overexpression of grape *DRT100* (Vv*DRT100-L*), in *Arabidopsis*, enhanced DNA repair under UV-B irradiation [72], suggesting for a specific role of VvDRT100-L in the repair/reduction of abasic sites and SSBs possibly arising from oxidative stress. The role of DRT100 in the context of seed development as well as the occurrence of DNA damage during this developmental transition is still unclear. However, we have evidence that oxidative damage may occur during early filling and seed dehydration in *P. vulgaris*. A strong accumulation (Log_2_ FC 4.85) of the 1-CYS PEROXIREDOXIN enzyme (EC 1.11.1.15/EC 1.11.1.7; A0A0B2RB28) from 10 DAA to 20 DAA and, at less extent from 30 to 40 DAA, was previously observed [21]. It is worth noting that 1-CYS PEROXIREDOXIN acts not only to relieve mild oxidative stresses but also as a molecular chaperone under severe stress conditions during seed germination and plant development [73]. 

Nucleotide excision repair has been described as the most versatile system for dealing with the DNA damage accumulated on desiccation and seed aging [74]. Our data show that the expression of different NER genes increases from 30 DAA to 40 DAA (Table S6, Figure 3), as a molecular signature of the seed desiccation process. It remains to be answered if this increase is triggered by the accumulation of DNA damage during this phase or if this is a protective mechanism by which the seed is being prepared to deal with this type of damages during early germination as suggested by [14].

### 4.2. Role of Chromatin and Chromatin Remodeling in DNA Damage Repair during Seed Development

Decompaction and subsequent restoration of the starting chromatin structure in conjunction with DDR create another level of complexity in genome maintenance regulation [75]. Chromatin consists mainly of nucleosomes, spherical octamers formed by two molecules of each of the four histone types H2A, H2B, H3, and H4 with 1.7 turns of DNA wrapped around their surface and sealed by H1 linker histones [13]. Several reports highlight that, besides their role in chromatin structure, histones play a major role in the regulation of gene transcription and DNA repair [13].

Numerous genes encoding for different HISTONE H2A proteins, GAMMA HISTONE VARIANT H2AX (G-H2A), H2B proteins, H4 and H3 were detected in our data. Generally, a strong decrease in their expression was detected in the transition from late embryogenesis to early filling (10 DAA to 20 DAA) suggesting that the enrolment of these proteins during embryogenesis was relevant. Considering the role of histone proteins in packaging DNA into chromatin, the synthesis of histones obviously needs to be tightly linked to DNA synthesis [76]. In the previous section, we discussed the role played by H2A.X, a variant of the H2A subunit conserved between eukaryotes, in the context of DNA damage signaling during early seed development. Increases in the expression of some members of the *HISTONE SUPERFAMILY PROTEIN* with strong homology with predicted members of *H3* (*H3.2*) and *H4* family were noticed on the transition from seed maturation to dehydration (30 DAA to 40 DAA, Table S4), which also marks the embryo entrance in the quiescent state characteristic of orthodox seeds, such as those of *P. vulgaris*. It is difficult to suggest a DDR role associated with the accumulation of those transcripts during seed dehydration but we cannot rule out that the accumulation of *H3/H4* transcripts, and therefore proteins, may influence the condensation state of the chromatin. In yeast, elevated histone levels are correlated with tighter chromatin structure, which leads to a transcriptional silencing of genome regions that might be involved in lifespan extension [77]. Based on these previous statements, we could speculate that high histone accumulation at 10 DAA and at 40 DAA could affect chromatin compactness during embryogenesis and dehydration, regulating gene transcription during seed development. In agreement with this hypothesis, the expression profiles of genes encoding for storage proteins such as the PAP85 CUPINS and RMLC-LIKE CUPINS PROTEIN families (see Table S3) and the profiles of some proteins implicated in carbohydrate metabolism, as the STARCH BRANCHING ENZYME (EC 2.4.1.18, Q9XIS5), GLUCOSE-1-PHOSPHATE ADENYLYLTRANSFERASE (EC 2.7.7.27; V7C329) [21] are opposite to the expression profiles of histone superfamily proteins.

Chromatin remodelers change the contacts between histones and DNA in the nucleosome, allowing them to arrange the distribution pattern of nucleosome spacing along the chromatin, thus playing a role in activation or repression of gene expression [78]. Previous works focused on seed germination have reported that chromatin remodelers act in concert with DNA repair machinery to maintain seed genome stability, essential for a successful germination [79]. Beside the changes on the expression of genes encoding for histones, we have also noticed interesting changes in the expression of genes encoding for chromatin remodelers as the *CHROMATIN REMODELING FACTOR17* (*CHR17*) and the *CHROMATIN REMODELING FACTOR CHD3* (*PKL*) during *P. vulgaris* seed development. The expression of *CHR17* was high at late embryogenesis (10 DAA) and subsequently decreases on early filling (20 DAA). Interestingly, the network analysis conducted for the same time points unveiled that *CHR17* bridges “DNA.synthesis/chromatin structure” and “RNA.regulation of transcription” MapMan functional categories (Figure 6A). CHR17 belongs to the imitation switch (ISWI)-type chromatin remodelers, which, in *Arabidopsis*, seem to be involved in sliding nucleosomes in gene bodies, with impact in gene transcription [78]. Based on these assumptions, it is not surprising to observe higher expression levels of *CHR17* during early seed development, when embryo and remaining seed compartment cells are actively dividing and differentiating. The expression of *PKL* also decreases in the transition from late embryogenesis to the early filling stage. This gene, a member of the CHD subfamily II with ATP-dependent chromatin remodeling activity [80], is involved in several key physiological processes such as floral transition mediated by *LEAFY* (*LFY*) expression in *Arabidopsis* [81]. PKL was also described as a repressor of the transcription factor (TF) LEAFY COTYLEDON1 (LEC1) upon embryogenesis [82,83]. LEC1 is part of the LEC1/ ABSCISIC ACID INSENSITIVE3 (ABI3), FUSCA3 (FUS3), and LEAFY COTYLEDON2 (LEC2) (AFL) regulatory network that regulates seed filling, maturation, and storage compound synthesis [82,83]. The *PKL* expression profile is opposite to the observed for storage proteins during seed development [21], such as LEGUMIN (F8QXP7) and PHASEOLIN (Q43632), an aspect that is in agreement with the regulatory function proposed for PKL on the LEC1/AFL regulatory network [82,83,84].

Most of the reports highlight that histone post-translational modifications (PTMs), e.g., acetylation and methylation of histone H3 and H4, are crucial in the regulation of DNA replication and transcription, as well as DNA repair [85]. Other post-translational modifications, as neddylation (Ubiquitin-NEDD8), appear to play a major role in the regulation of developmental processes during embryogenesis in plants [86,87]. We detected a high expression level of *RUB1*, a gene encoding a ubiquitin-NEDD8-like protein transition at late embryogenesis (10 DAA) decreasing afterward (Table S3). In our previous proteomic analysis [21], we speculated that several metabolic pathways acting during early SD could be regulated via neddylation, since the accumulation of ubiquitin-NEDD8-like protein RUB2 was high at 10 DAA. Evidence from a study conducted in human cells found that histone H4 was polyneddylated in response to DNA damage and NEDD8 was conjugated to the N-terminal lysine residues of H4 [88]. Nevertheless, more studies are needed to understand the role of neddylation in modulating DDR responses during seed development.

## 5. Conclusions

Mechanisms of genome integrity maintenance are active during seed development in *Phaseolus vulgaris*. The results show that most genes related to the DNA damage response and chromatin remodeling are more expressed during late embryogenesis when cell division and differentiation occurs. Among them, the expression of the *PCNA1*, the *RPA* and the *RUB1* were found highly expressed at 10 DAA. Genes involved in chromatin structure and remodeling, such as *H2AX*, the *CHR17* and the *CHD3* were also found highly expressed at the same time point. This suggests relevant mechanisms acting, possibly involved in the modulation of DNA damage response arising from rapid cell division and high metabolic activity characteristic of embryo morphogenesis.

Evidence of the activation of seed protection mechanisms during seed desiccation was also found. An upregulation of *SOG1*, *NAP1*, *MRE11*, *NBS1* and *RAD50*, implicated in the sensing of DNA double-strand breaks and signal transduction, was observed at 30 DAA, suggesting that genome integrity is also challenged at the onset of seed dehydration. An increase of the expression of genes associated with nucleotide excision repair was also noticed in the transition from 30 DAA to 40 DAA. An increase in the expression of some *HISTONE SUPERFAMILY PROTEIN* members was also observed during seed desiccation, possibly affecting chromatin compactness while regulating gene transcription.

Despite the evidence provided by this study, there are still open questions that need to be addressed. It remains to be understood the molecular mechanisms that are underlying the temporal regulation of gene expression seen during seed development. Although it could be interesting to investigate DDR and chromatin remodeling mechanisms very early in seed development, more studies need to be focused on seed protection mechanisms activated during desiccation, since they could play a major role in seed viability. In due time, the molecular resources generated in this study would provide new targets that could be used to breed seeds with improved seed viability.

## Figures and Tables

**Table 1:** List of selected genes and description of the primers and probes constructed to be used for digital PCR.

GENE ID	Gene Symbol	Description	Primer Forward Sequence (5’‒3’)	Primer Reverse Sequence (5’‒3’)	MGB Probe Sequence (5’‒3’)	Dye
Phvul.003G027100	*SOG1*	SUPPRESSOR OF GAMMA RESPONSE 1	TGGGACAGTGAGTCACAGAA	GAGCATAAACAGAAAGACCAGGAT	CTGGGAGACTGGCTGTGGAGGAGAT	FAM^TM^
Phvul.006G003000	*ATM*	ATAXIA-TELANGIECTASIA MUTATED	TGGACTCAGATCAGGCATTGA	CACCAAAATCAGTGTCACCTCTT	AGCAGGCGGCAATGGATGTGGTT	FAM^TM^
Phvul.006G095800	*G-H2AX*	GAMMA HISTONE VARIANT H2AX	GGTGAGGAATGATGAGGAACTG	ACTCTTGTGAAGCAGATCCAA	CCGTTCGCAATGGTGACAGACCCC	FAM^TM^
Phvul.005G085700	*MRE11*	DNA REPAIR AND MEIOSIS PROTEIN (MRE11)	CCACCTCGGGTATATGGAGAA	AAATCTCTTCAAAGGCGTGGAA	ATGAGGTGCGCCGCCACGACT	FAM^TM^
Phvul.001G266800	*RAD50*	DNA REPAIR-RECOMBINATION PROTEIN (RAD50)	TGATGGTATGCGGCAAATGTTT	GCTGAACTAGTAGCCTTCACTCTT	TGCCCTTGCTGTGAACGCCCT	VIC^TM^
Phvul.008G242800	*NBS1*	NIJMEGEN BREAKAGE SYNDROME 1	CAAGGTTGATGATAATGAAACTGGAA	GAGTGTGTGCCTTTCTGAAACAT	TGCTGTCTGGTGCAGTGCTTACGCT	VIC^TM^
Phvul.001G204900	*WEE1*	WEE1 KINASE HOMOLOG	CTCATTCCTCTCAACCAACCA	GTGAGCACAACGCACGAT	CCTCCGTTTCCTGCTTCCAGAACCC	VIC^TM^
Phvul.003G135500	*NAP1;2*	NUCLEOSOME ASSEMBLY PROTEIN 1;2	CTTTCACCTCTGCAATGAGTAAC	CCGCTCTATTTTCCTCGTTGA	AGGACACCTTCAACGTCGCCGATCT	VIC^TM^

**Table 2:** Characterization of the constructed massive analysis of 3’-cDNA ends (MACE) libraries. Total raw, cleaned, and mapped reads of each sequenced sample on the Illumina Hiseq2000 are described. Percentage of mapped reads was calculated using the number of mapped reads/cleaned reads. DAA: days after anthesis.

Pooled Sample ID	Raw Reads	Cleaned Reads	Mapped Reads	% Mapped Reads
10 DAA	10880000	8000000	7818197	97.73%
20 DAA	10767653	7876850	7633075	96.91%
30 DAA	11648490	8477795	8296129	97.86%
40 DAA	8255161	5772840	5518643	95.60%

## Supplementary Materials

The following are available online at http://www.mdpi.com/2073-4425/9/10/463/s1. Figure S1: Correlation between the Log_2_ expression values from the transcriptomic and dPCR datasets, for all candidate genes at all time points; Figure S2: Schematic representation of the double-strand break (DSB) sensing mechanism in plants (adapted from [3,7,61]). Table S1: Primer/probe details used for dPCR. Table S2: Percentage and number of expressed genes included in MapMan functional categories. Table S3: All EGs with the respective Gene ID, Description, normalized and raw read count and Log_2_FC. Table S4: All EGs that were functionally categorized as “DNA” using MapMan; Table S5: Blast results for histone family genes. Table S6: List of EGs identified as associated with DNA repair mechanisms (classification based on [4]).
